# The C-Type Lectin Receptor SIGNR3 Binds to Fungi Present in Commensal Microbiota and Influences Immune Regulation in Experimental Colitis

**DOI:** 10.3389/fimmu.2013.00196

**Published:** 2013-07-16

**Authors:** Magdalena Eriksson, Timo Johannssen, Dorthe von Smolinski, Achim D. Gruber, Peter H. Seeberger, Bernd Lepenies

**Affiliations:** ^1^Department of Biomolecular Systems, Max Planck Institute of Colloids and Interfaces, Potsdam, Germany; ^2^Department of Biology, Chemistry and Pharmacy, Institute of Chemistry and Biochemistry, Freie Universität Berlin, Berlin, Germany; ^3^Department of Veterinary Pathology, Freie Universität Berlin, Berlin, Germany

**Keywords:** SIGNR3, C-type lectin receptor, host innate immunity, colitis, carbohydrate recognition, microbiota, fungi

## Abstract

Inflammatory bowel disease is a condition of acute and chronic inflammation of the gut. An important factor contributing to pathogenesis is a dysregulated mucosal immunity against commensal bacteria and fungi. Host pattern-recognition receptors (PRRs) sense commensals in the gut and are involved in maintaining the balance between controlled responses to pathogens and overwhelming innate immune activation. C-type lectin receptors (CLRs) are PRRs recognizing glycan structures on pathogens and self-antigens. Here we examined the role of the murine CLR specific intracellular adhesion molecule-3 grabbing non-integrin homolog-related 3 (SIGNR3) in the recognition of commensals and its involvement in intestinal immunity. SIGNR3 is the closest murine homolog of the human dendritic cell-specific intercellular adhesion molecule-3-grabbing non-integrin (DC-SIGN) receptor recognizing similar carbohydrate ligands such as terminal fucose or high-mannose glycans. We discovered that SIGNR3 recognizes fungi present in the commensal microbiota. To analyze whether this interaction impacts the intestinal immunity against microbiota, the dextran sulfate sodium-induced colitis model was employed. SIGNR3^−/−^ mice exhibited an increased weight loss associated with more severe colitis symptoms compared to wild-type control mice. The increased inflammation in SIGNR3^−/−^ mice was accompanied by a higher level of TNF-α in colon. Our findings demonstrate for the first time that SIGNR3 recognizes intestinal fungi and has an immune regulatory role in colitis.

## Introduction

Inflammatory bowel disease (IBD) is a condition of acute and chronic inflammation and can be classified in ulcerative colitis (UC) and Crohn’s disease (CD). UC mainly affects the mucosal lining of the colon and rectum, whereas inflammation in CD occurs throughout the whole intestinal wall and may affect the whole gastrointestinal tract (1). The mechanisms underlying these severe diseases are poorly understood. However, epidemiological and genetic studies in patients along with IBD-related animal models suggest that a combination of genetic susceptibility, environmental factors, and an altered immune response contribute to pathogenesis (2). In particular, an imbalance of the mucosal immunity against intestinal microbiota plays an essential role in colitis induction (3, 4).

While the role of T cells in gut inflammation has been studied in detail (5–7), there are limited insights in the involvement of innate immunity in colitis. However, certain mediators of innate immunity such as members of the TNFR superfamily (8), co-stimulatory molecules (7, 9), or pattern-recognition receptors (PRRs) (3, 10, 11) are involved in immune regulation during colitis. The interaction of intestinal dendritic cells (DCs) with commensals influences the fine balance between tolerance and immune activation against commensals (12–14). DCs recognize danger signals through PRRs that mediate binding and uptake of pathogens and initiate signaling pathways (10, 15–17). Intestinal DCs from IBD patients express a higher level of the DC activation marker CD40 than healthy controls (18). In addition, the expression of certain PRRs, such as Toll like receptors (TLRs), is upregulated on intestinal DCs in IBD patients (18, 19). Recognition of microflora antigens and activation of TLRs are crucial for maintaining intestinal homeostasis since mice lacking the adaptor protein MyD88 exhibit severe weight loss along with epithelial damage upon colitis induction (20, 21). Members of the Nucleotide oligomerization domain (NOD)-like receptor family such as nucleotide oligomerization domain 2 (NOD2) are involved in the intestinal immune response by regulating the initiation and progression of colitis (10, 11, 22, 23).

C-type lectin receptors (CLRs) are transmembrane PRRs recognizing carbohydrate structures present on pathogens or self-antigens often in a Ca^2+^-dependent manner (16). The carbohydrate recognition specificities as well as the cytoplasmic signaling motifs differ among the different CLR subfamilies (16, 24). Recent studies indicate that CLRs are involved in gastrointestinal inflammation and to date, four CLRs [Dectin-1, MGL1, mannose binding lectin (MBL), and SIGNR1] have been shown to influence the development of murine colitis (25–28). The importance of the CLR Dectin-1 in intestinal immunity was recently demonstrated (28). Dectin-1 recognizes commensal fungi and mice lacking Dectin-1 exhibited an increased susceptibility to chemically induced colitis. A polymorphism in the human Dectin-1 gene (CLEC7A) is associated with medically refractory UC indicating the relevance of Dectin-1 for intestinal inflammation (28).

The human CLR dendritic cell-specific intercellular adhesion molecule-3-grabbing non-integrin (DC-SIGN) specifically interacts with fucose- and mannose-containing glycans (29, 30). Of the eight murine homologs of DC-SIGN (31, 32), specific intracellular adhesion molecule-3 grabbing non-integrin homolog-related 3 (SIGNR3/CD209d) has the most similar biochemical characteristics to the human receptor. Similar to DC-SIGN, SIGNR3 recognizes terminal fucose, galactose, *N*-acetylgalactosamine residues as well as fungal mannan structures (32–34). Mice lacking SIGNR3 are more susceptible to tuberculosis indicating the involvement of SIGNR3 in early pulmonary resistance to *M. tuberculosis* (35). In a recent study, SIGNR3 was reported to be involved in regulating the cellular immune response to *Leishmania infantum* infection via LTB4-dependent reduction of IL-1β production (36).

To date, the role of SIGNR3 in inflammation and autoimmunity has not yet been elucidated. Thus, we analyzed the involvement of SIGNR3 in the recognition of intestinal microbiota and its involvement in intestinal inflammation. We show SIGNR3-dependent binding to microbiota that can be inhibited by mannan. Further analyses demonstrate that SIGNR3 binds indigenous fungi. Consistently, SIGNR3^−/−^ mice exhibited a higher susceptibility to dextran sulfate sodium (DSS)-induced colitis as demonstrated by increased weight loss and aggravated colitis symptoms compared to wild-type mice. Exacerbation of DSS colitis in SIGNR3^−/−^ mice was accompanied by an increased TNF-α production in the colon suggesting that fungal recognition by SIGNR3 contributes to intestinal homeostasis. In conclusion, we show for the first time that SIGNR3 recognizes fungi present in the intestinal microbiota, thus plays an immune regulatory role in colitis.

## Materials and Methods

### Production and characterization of CLR-hFc proteins

C-type lectin receptors were expressed as soluble proteins fused to the Fc part of human IgG1. The extracellular regions of the murine SIGNR3, MGL1, and dendritic cell immunoactivating receptor (DCAR) were amplified by PCR and ligated into the expression vector pFUSE-hIgG1-Fc2 (Invivogen, Toulouse, France; Figure S1A in Supplementary Material). Proteins were transiently expressed using the FreeStyle™ MAX CHO Expression System (Life technologies, Darmstadt, Germany). Characterization and functionality test of the SIGNR3-hFc protein was performed by western blot analysis and ELISA (Figures S1A–D in Supplementary Material).

### Binding studies

To analyze the interaction of SIGNR3 with commensal microbes, heat inactivated intestinal microbes were used. Fresh stool from colon of C57BL/6 wild-type mice was inactivated at 65°C for 2 h and diluted in PBS to an OD_600_ of 0.6. Microbiota were coated on 96-well high binding plates (Greiner, Frickenhausen, Germany) overnight. After blocking with 1% BSA, 10 μg/ml of SIGNR3-hFc, DCAR-hFc, or hFc (Merck Millipore, Darmstadt, Germany) were incubated in lectin binding buffer (50 mM HEPES, 5 mM MgCl_2_, 5 mM CaCl_2_, pH 7.4) or EDTA buffer (25 mM EDTA, 50 mM HEPES, pH 7.4) at RT for 2 h. Washing steps were performed with either lectin binding buffer or EDTA buffer. Coated zymosan incubated with SIGNR3-hFc was used as a positive control. Binding was detected by an alkaline phosphatase-conjugated goat anti-hFc antibody (Dianova, Hamburg, Germany) and developed using *p*-nitrophenyl phosphate (Thermo Scientific, Rockford, IL, USA).

To further characterize the microbial component recognized by SIGNR3, stool microbes were labeled with SYTO^®^ 61 red fluorescent nucleic acid stain (Life Technologies, Darmstadt, Germany) at 2.5 μM in PBS at RT for 30 min and washed three times in PBS. Labeled microbiota were incubated with 1 μg/ml CLR-hFc fusion protein in lectin buffer supplemented with 0.5% BSA at 4°C for 1 h. After two additional washing steps, samples were incubated with PE-conjugated goat anti-hFc antibody (Dianova). Carbohydrate specificity of the binding was analyzed by preincubation of SIGNR3-hFc with mannan at a concentration of 0.01–10 mg/ml at 4°C for 30 min. Commensal fungi were detected by staining the samples with FITC-conjugated rabbit anti-*C. albicans* (Meridian Life Science Inc., Memphis, TN, USA) at 4°C in the dark for 30 min. This antibody cross-reacts with other yeasts as described by the manufacturer and as previously reported (28). Samples were washed three times and analyzed using a FACSCanto II flow cytometer (BD Pharmingen, Heidelberg, Germany). Data were analyzed using FlowJo analysis software (Tree Star Inc., Ashland, OR, USA).

### Chemically induced colitis

Mice were housed under special pathogen free (SPF) conditions and were provided food and water *ad libitum*. The mouse strain 031934-UCD, SIGNR3 KO/Mmcd was provided by the NIH-sponsored Mutant Mouse Regional Resource Center (MMRRC) National System. Genotyping of the SIGNR3-gene in wild-type and SIGNR3^−/−^ mice was performed using a protocol provided by the Consortium for Functional Glycomics (Figure S2 in Supplementary Material). Colitis was induced in 8–11 week old female SIGNR3-deficient mice or the respective wild-type C57BL/6 control mice by adding 3% DSS salt (reagent grade, 35–50 g/mol, MP Biomedicals, Illkirch, France) to the drinking water for eight consecutive days. Body weight was recorded daily. On day seven, mice were sacrificed and a disease activity index (DAI) was assigned to each mouse (Table 1) as described previously (27), modified from (37). Briefly, a score of 0–4 was assigned to each mouse depending on stool consistency, presence of blood in feces, and weight loss. Blood in feces was detected with Greegor’s modified Guaiak test (Haemoccult, Beckman Coulter, Galway, Ireland) according to the manufacturer’s instructions.

**Table 1:** **Scoring for disease activity index**.

Score	Blood in feces	Stool consistency	Weight loss (%)
0	No blood detected	Normal	0
1			1–3
2	Blood detected	Loose stool	3–6
3			6–9
4	Gross blood detected	Diarrhea	>9

Animal experiments were performed in strict accordance with the German regulations of the Society for Laboratory Animal Science (GV-SOLAS) and the European Health Law of the Federation of Laboratory Animal Science Associations (FELASA). The protocol was approved by the Landesamt für Gesundheit und Soziales (LAGeSo) Berlin (Permit Number: G0052/10). All efforts were made to minimize suffering.

### Histology

Whole colon of each mouse was prepared as a Swiss roll in an embedding cassette and immersion fixed with 4% buffered formalin. Samples were embedded in paraffin, sectioned at 4 μm thickness and stained with hematoxylin and eosin (H&E). Sections were evaluated histopathologically in a blinded manner. Each colon was divided into three segments of identical lengths and each part was graded according to the degree of infiltration of inflammatory cells and mucosal erosion/ulceration (Table 2).

**Table 2:** **Scoring system for histological evaluation of intestinal lesions**.

Score	Infiltration of inflammatory cells	Mucosal erosion/ulceration
0	None	None
1	Mild	Mild
2	Moderate	Moderate
3	Severe	Severe

### Colon homogenization

Colon was homogenized using a modified Greenberger lysis buffer containing 300 mM NaCl, 15 mM Tris, 2 mM MgCl2, 0.5% Triton X-100, Protease-inhibitor X, and Protease-inhibitor HP (Serva Electrophoresis, Heidelberg, Germany) using an IKA T10 homogenizer (IKA-Werke GmbH, Staufen, Germany). Protein concentration was determined by the Pierce BCA Protein Assay Kit (Thermo Scientific, Rockford, IL, USA) with BSA as a standard.

### Cytokine measurement

The measurement of cytokines in colon homogenate was performed using cytometric bead array (BD Biosciences) as described by the manufacturer. Analysis was performed with a FACSCanto II flow cytometer (BD Biosciences) and the FCAP Array™ software (BD Bioscience). The following cytokines were measured: IL-6, IL-10, TNF-α, IL-12p70, and IL-1β.

### Statistical analysis

Statistical analyses were performed with the unpaired Student’s *t*-test using the Prism program (GraphPad Software, La Jolla, CA, USA). For analysis of the body weight, a mixed linear model was used taking multiple measurements per mouse into account and the structure of these measurements was assumed to be autoregressive. In addition to the parameter “group,” “day” was also a parameter in the model. The analysis of the mixed linear model was performed using SAS software version 9.3 (SAS Institute Inc., Cary, NC, USA). A *p*-value of *p* < 0.05 was considered statistically significant.

## Results

### SIGNR3 binds to fungi present in commensal microbiota

**Figure 1:**
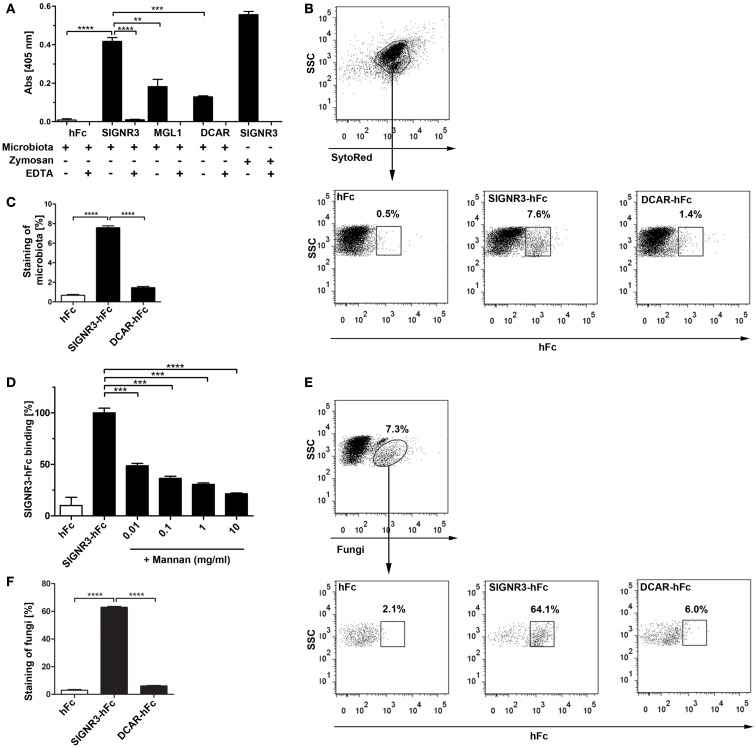
**Binding of SIGNR3 to fungi present in commensal microbiota**. **(A)** ELISA analysis of binding of CLR-hFc fusion proteins to heat inactivated intestinal microbiota demonstrated significant binding of SIGNR3-hFc. Binding of SIGNR3-hFc to zymosan, and MGL1-hFc to colon microbiota were used as positive controls. If indicated, incubation and washing was performed in 25 mM EDTA buffer to analyze Ca^2+^-dependency of the interaction. Results are representative of three independent experiments. **(B)** Representative dot plots of SytoRed stained gut microbes (APC^+^) incubated with hFc, SIGNR3-hFc, or DCAR-hFc. Detection with a PE-labeled goat anti-hFc antibody demonstrates binding of SIGNR3-hFc to gut microbes. **(C)** Corresponding statistical analysis of flow cytometry data representative from three independent experiments. **(D)** The interaction of SIGNR3-hFc and microbes was inhibited by preincubation with *S. cerevisiae*-derived mannan in the concentrations of 0.01–10 mg/ml. Data are representative of two independent experiments. **(E)** Staining with a FITC-labeled anti-*C. albicans* antibody reported to cross-react with other fungi revealed about 7% of the microbes were fungi. Staining of cells with fusion proteins demonstrated that SIGNR3-hFc recognizes>60% of the commensal fungi in the colon. **(F)** Statistical analysis of commensal fungi detected by SIGNR3- and DCAR-hFc as determined by flow cytometry. Shown are data and gating representative for three independent experiments. **(A–F)** Data are presented as mean + SEM. The *p*-values were determined using unpaired Student’s *t*-test. **p < 0.01, ***p < 0.001, ****p < 0.0001.

The CLR Dectin-1 recognizes commensal fungi and plays a crucial role in intestinal immunity (28). To investigate whether SIGNR3 contributes to the recognition of intestinal microbiota, we generated a SIGNR3-hFc fusion protein (Figure S1 in Supplementary Material) and determined binding of this fusion protein to heat inactivated gut microbes using an ELISA-based binding assay. Notably, a significantly higher interaction of SIGNR3-hFc to intestinal microbiota was detected compared with MGL1-hFc and DCAR-hFc produced in the same way, or human Fc (hFc) alone, indicating specificity of the SIGNR3-microbiota interaction. This interaction was almost completely abolished when samples were incubated in the presence of 25 mM EDTA, indicating a Ca^2+^-dependent interaction between SIGNR3 and intestinal microbiota (Figure 1A). Furthermore, SIGNR3-hFc binding to microbiota was concentration-dependent (data not shown). MGL1 was reported to bind *Streptococcus spp*. and *Lactobacillus spp*. present in the gut microbiota (25). Noteworthy, the degree of interaction of SIGNR3-hFc with commensal microbiota observed in this study even exceeded that of MGL1-hFc, suggesting that SIGNR3 might also be involved in intestinal recognition of microbes. In contrast, DCAR-hFc displayed only marginal interaction with stool microbes.

To further examine the binding of SIGNR3 to the microbiota, we characterized this interaction by flow cytometry. Fusion proteins were incubated with stool microbes and detected using a fluorescently labeled anti-hFc antibody. These binding studies revealed that SIGNR3-hFc bound to about 8% of the microbes (Figures 1B,C). Consistent with our results obtained by ELISA, this interaction was significant compared with DCAR-hFc and the specificity control hFc (Figure 1C). Furthermore, the interaction of SIGNR3-hFc and the microbes was blocked by mannan derived from *S. cerevisiae* in the concentration of 0.01–10 mg/ml indicating specific carbohydrate recognition by SIGNR3 (Figure 1D).

To examine which subclass of microbes was recognized by SIGNR3, co-staining with an antibody against *C. albicans* was performed. This antibody cross-reacts with other yeasts as described by the manufacturer and as previously reported (28) and stained about 7% of the gut microbiota (Figure 1E). When microbiota were co-stained with the anti-fungal antibody and SIGNR3-hFc,>60% of fungi detected by the antibody were also stained by SIGNR3-hFc (Figures 1E,F). In contrast, DCAR-hFc bound only marginally to fungi recognized by the anti-fungal antibody (Figures 1E,F). This finding indicates that SIGNR3-hFc binds specifically to a majority of intestinal fungi present in the murine commensal microbiota.

### SIGNR3 deficiency exacerbates DSS-induced colitis

**Figure 2:**
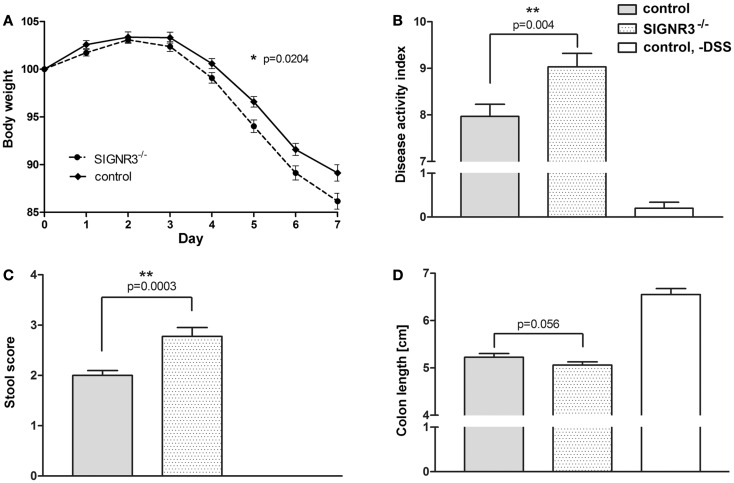
**Wild-type and SIGNR3^−/−^ mice were fed 3% DSS supplemented to the drinking water for seven consecutive days (*n* = 31 for SIGNR3^−/−^ mice, *n* = 30 for wild-type mice)**. **(A)** Weight was recorded daily. On day 7, disease activity index **(B)**, stool score **(C)**, and colon length **(D)** were measured. SIGNR3^−/−^ mice displayed an increased susceptibility to DSS colitis as indicated by a more severe weight loss and exacerbated colitis symptoms. A summary of four independent experiments is shown. Data are expressed as mean + SEM. The *p*-values were determined using a mixed linear model **(A)** and unpaired Student’s *t*-test **(B–D)**. Significance is indicated by asterisks (*), *p < 0.05, **p < 0.01, ***p < 0.001.

Since SIGNR3 binds to fungal populations in the gut, we hypothesized that this interaction might play a role in intestinal immune homeostasis as has been described for Dectin-1 (28). To test this hypothesis, DSS colitis was induced in SIGNR3^−/−^ mice (for genotyping, see Figure S2 in Supplementary Material) and the respective wild-type control mice. Both wild-type and SIGNR3^−/−^ mice lost significantly weight from day 4 on and both strains displayed marked colitis symptoms on day 7 (Figures 2A–D). However, the weight loss at day 4–7 was significantly more pronounced in DSS-treated SIGNR3^−/−^ mice compared to wild-type mice (Figure 2A). In addition, the DAI was markedly increased in SIGNR3^−/−^ mice (Figure 2B) indicating that SIGNR3 deficiency led to an exacerbation of colitis. Blood was present in stool of almost all mice from both strains treated with 3% DSS. However, severe diarrhea (score 4) was present more often in SIGNR3^−/−^ mice than in wild-type mice resulting in a significantly increased stool score (Figure 2C). No such symptoms or weight loss were present in untreated SIGNR3^−/−^ mice or untreated wild-type mice (data not shown). The colon was significantly shorter in all mice treated with DSS compared to colon from untreated wild-type mice (Figure 2D). No significant difference was observed between SIGNR3^−/−^ mice and wild-type mice though colon shortening tended to be more pronounced in SIGNR3^−/−^ mice.

**Figure 3:**
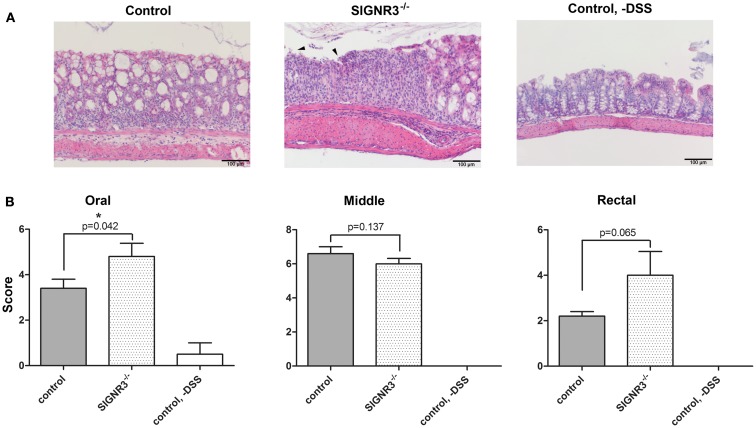
**After 7 days of DSS treatment, mice were sacrificed and paraffin sections of the colon of each mouse were prepared and stained with hematoxylin and eosin (H&E)**. A histopathological evaluation of each section was performed in a blinded manner. **(A)** H&E stained sections from colon of C57BL/6 wild-type, and SIGNR3^−/−^ mice at day 7, and untreated control mice. **(B)** Each colon was divided into three segments of identical length (oral, middle, rectal) and each segment of the colon was analyzed separately. The degree of infiltration of inflammatory cells and mucosal erosion/ulceration was graded from none (score 0) to mild (score 1), moderate (score 3), or severe (score 4) (*n* = 5). Data are expressed as mean + SEM. The *p*-values were determined using unpaired Student’s *t*-test. Arrows show examples of mucosal ulceration. Significance is indicated by asterisks (*), *p < 0.05.

Histological analysis of H&E stained colon tissue revealed a severe, acute, multifocal to coalescent, partly transmural UC in wild-type and SIGNR3^−/−^ mice treated with DSS. Both groups had severe submucosal edema and adjacent steatitis. However, the percentage of altered tissue was increased in SIGNR3^−/−^ mice compared to wild-type mice (Figure 3A). Interestingly, tissue damage and ulceration was exacerbated in SIGNR3^−/−^ mice both in the oral and rectal part of the colon, whereas the middle part of the colon was equally affected in SIGNR3^−/−^ and wild-type mice (Figure 3B). Thus, histological evaluation confirmed the higher susceptibility of SIGNR3^−/−^ mice to DSS-induced colitis.

### SIGNR3 deficiency leads to an increased TNF-α production

**Figure 4:**
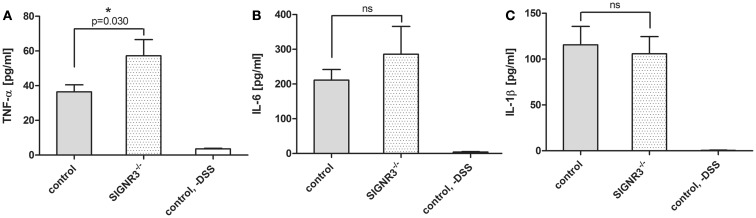
**Cytokines in colon homogenates from wild-type and SIGNR3^−/−^ mice fed with 3% DSS were analyzed by cytometric bead array**. The levels of TNF-α **(A)**, IL-6 **(B)**, and IL-1β **(C)** were measured (*n* = 8 for DSS-treated SIGNR3^−/−^ and wild-type mice, *n* = 5 for untreated wild-type mice). Data are expressed as mean + SEM. The *p*-values were determined using unpaired Student’s *t*-test. Significance is indicated by asterisks (*), *p < 0.05.

To address the mechanism that leads to the increased inflammation in SIGNR3^−/−^ mice during DSS colitis, the local cytokine response in the colon was analyzed. Pro-inflammatory cytokines such as TNF-α, IL-1β, and IL-6 were detectable in both SIGNR3^−/−^ and wild-type mice upon induction of DSS colitis (Figure 4). The concentrations of all tested cytokines were below the detection level in untreated wild-type (Figure 4) and SIGNR3^−/−^ mice (data not shown). Interestingly, the TNF-α level was significantly higher in the colon of SIGNR3^−/−^ mice during DSS colitis (Figure 4A). To analyze whether the production of immune regulatory cytokines was affected by SIGNR3 deficiency, IL-10 levels in colon homogenates were determined. However, levels were below the detection level in both SIGNR3^−/−^ and wild-type mice upon colitis induction (data not shown) thus rendering a role of IL-10 unlikely. In conclusion, the lack of SIGNR3 binding to fungi is accompanied by an increased TNF-α production, indicating a role for SIGNR3 in intestinal immune homeostasis.

## Discussion

We report that SIGNR3 recognizes commensal fungi and is involved in intestinal immune homeostasis during colon inflammation. To date, SIGNR3 has been reported to bind to *M. tuberculosis* (35) and *C. albicans* as well as the fungal cell wall components zymosan and mannan (34). Consistently, the binding of SIGNR3-hFc to commensal microbes was inhibited by yeast-derived mannan. Mannan constitutes the outer cell wall layer in nearly all fungi (38) and is recognized by several PRRs such as TLR4, and the CLRs Mincle and Dectin-2, mediating anti-fungal immune responses (39, 40). We thus postulated that SIGNR3 might be involved in the recognition of intestinal fungi. Indeed, SIGNR3-hFc bound to the majority of present fungi as illustrated by co-localization with an anti-fungal antibody. These findings show that SIGNR3 recognizes fungi in the microbiota presumably by binding to mannose-rich structures. This recognition of fungi is consistent with previous studies reporting recognition of fungi-derived mannan, *C. albicans* (41, 42) and *S. cerevisiae* (43) by human DC-SIGN. Activation of DC-SIGN by fungi leads to activation of the raf-1 kinase, acetylation of NF-kB and thereby an increase of TLR-induced IL-10 production (44). An increased population of DC-SIGN^+^ DCs producing the cytokines IL-12 and IL-18 was detected in colonic mucosa of patients with CD (45) suggesting the involvement of this receptor in intestinal homeostasis. This report together with the present study suggests that DC-SIGN might be involved in the regulation of intestinal inflammation.

The discovered recognition of commensal fungi by SIGNR3 highlights that certain CLRs are involved in anti-fungal responses in the gut. Binding of fungal mannan to Dectin-2, Mannose receptor (MR), DC-SIGN, and Mincle, or the interaction of fungal β-glucan with Dectin-1, induces intracellular signaling important for anti-fungal immunity (40). For example, recognition of *Malassezia* antigens by Dectin-1 and Mincle activates TNF-α and IL-10 production (46, 47). To date, Dectin-1 is the only CLR reported to bind to fungi present in the gut microbiota (28). Iliev et al. showed that the direct interaction between commensal fungi and the CLR Dectin-1 influences the intestinal immune response during colitis (28). To analyze whether the interaction of SIGNR3 and commensal fungi also influences the intestinal immune response, we employed the DSS colitis model. This model has the advantage that commensal microbes that are normally restricted to the outer mucosal layer can penetrate the inner mucosal layer. As a consequence, microbes interact with epithelial cells and are also recognized by local immune cells which leads to the initiation of an inflammatory response against the commensals (48, 49). Thus, this model is suitable for analyzing the interplay of innate immunity and the commensal microbiota (50). Indeed, after colitis induction, SIGNR3^−/−^ mice exhibited a more severe weight loss and exacerbated colitis symptoms such as severe diarrhea, cell infiltration into the colon, and mucosal ulceration compared to wild-type mice, indicating an immune regulatory role of SIGNR3 in the gut.

Previous studies of mice lacking other PPRs, e.g., TLRs or NOD-like receptors, which recognize microbial antigens, show the involvement of PRRs in murine colitis (11, 20, 51). Alike the SIGNR3^−/−^ mice in our study, mice lacking NOD2 are more susceptible to colitis (23). Binding to the microbial ligand muramyl dipeptide activates NF-kB signaling pathways (52). However, the administration of NOD2 ligands to wild-type mice ameliorates colitis symptoms (53) and NOD2 is involved in inducing a tolerogenic environment by up-regulation of IL-10 expression and proliferation of regulatory T cells (53).

Similar to our study, mice lacking Dectin-1 are more susceptible to chemically induced colitis. This disease exacerbation is caused by an altered immune response to fungal microbiota (28). The portion of pathogenic fungal populations such as *Candida* and *Trichosporon* is increased in Dectin-1^−/−^ mice further demonstrating the involvement of fungi and this CLR in the intestinal immune regulation (28). Worse colitis symptoms along with increased anti-fungal antibody titers were also found in MBL^−/−^ mice suggesting a role in the recognition of fungal commensals (26). Another example of a CLR influencing the intestinal immunity is the protective role of MGL1 in colitis demonstrated by more severe inflammation in MGL1^−/−^ mice during colitis (25). In contrast to these studies, mice lacking SIGNR1 exhibited an ameliorated form of murine colitis, with a reduction in the disease severity, colon damage, and levels of the pro-inflammatory cytokines IL-1β, TNF-α, and IL-6 (27). These reports together with the present study demonstrate that certain CLRs influence the inflammatory responses in murine colitis. We here report exacerbated symptoms detected in SIGNR3^−/−^ mice during colitis. Since certain myeloid CLRs signal via common adaptors leading to Syk recruitment (24), it is possible that the lack of SIGNR3 might be partially compensated by other CLRs involved in the regulation of the intestinal homeostasis. Thus, symptoms might even be more pronounced in mice lacking multiple CLRs. However, the findings that one single CLR has a marked influence on colitis pathogenesis points at an indispensable role of SIGNR3 in murine colitis.

Upon colitis induction, a significantly increased level of TNF-α was present in the colon of SIGNR3^−/−^ mice compared to wild-type mice. TNF-α plays a key role in IBD (54, 55) and administration of TNF-α-neutralizing antibodies is an approved medical therapy for IBD patients (56). Notably, mice lacking the CLR Dectin-1 also display higher TNF-α levels in the colon after induction of colitis (28). Similar to Dectin-1, SIGNR3 has an intracellular hemITAM and is suggested to signal via Syk and activation of either of these CLRs is reported to induce TNF-α production (24, 35). This increased TNF-α production in SIGNR3^−/−^ mice suggests that the recognition of commensal fungi by SIGNR3 is required for regulating homeostasis during intestinal inflammation. This is similar to a very recent report on *L. infantum* infection where DC-SIGN and SIGNR3 were reported to play a regulatory role in *L. infantum* infection, and were suggested to be important for the homeostasis in tissue areas with high microbial content (36).

To date, a functional relevance of SIGNR3 has only been described during *M. tuberculosis* infection (27). In the present study, we show for the first time that SIGNR3 is involved in the recognition of fungi present in the intestinal microbiota. Our findings indicate that SIGNR3 is an important regulator of immunity to fungal commensals in the colon, thus highlighting the importance of this CLR in commensal recognition and maintenance of intestinal immune homeostasis. Targeting of SIGNR3 or DC-SIGN might constitute a novel strategy to influence cellular responses to commensal fungi and thereby modulate chronic intestinal inflammation (57).

## Conflict of Interest Statement

The authors declare that the research was conducted in the absence of any commercial or financial relationships that could be construed as a potential conflict of interest.

## Supplementary Material

The Supplementary Material for this article can be found online at http://www.frontiersin.org/Molecular_Innate_Immunity/10.3389/fimmu.2013.00196/abstract

Supplementary Figure S1 **Expression and functionality of recombinant SIGNR3-hFc**. **(A)** Expression cassette used for the production of SIGNR3-hFc. The cDNA encoding for the extracellular part of SIGNR3 (SIGNR3 ECD) was amplified using the following primers: SIGNR3 forward 5′-GAATTCCATGCAACTGAAGGCTGAAG-3′ and SIGNR3 reverse 5′ AGATCTTTTGGTGGTGCATGATGAGG-3′. The product was fused in frame to the Fc region of human IgG1. Expression was driven by a hEF1-HTLV promoter and secretion into the culture supernatant was mediated by an external IL2 signal sequence (IL2ss). **(B)** Western blot analysis of recombinant SIGNR3-hFc. Marker (PageRuler Plus Prestained Protein ladder, Thermo scientific) and 100 ng human Fc (hFc) or SIGNR3-hFc were separated by SDS PAGE, transferred to a nitrocellulose membrane, and detected using an HRP conjugated goat anti-hFc antibody. The calculated molecular weight of the SIGNR3-Fc fusion protein is 44.7 kDa. MGL1-hFc and DCAR-hFc were produced in the same way using target-specific primers. **(C,D)** Binding of SIGNR3-hFc to immobilized mannan **(C)** or zymosan **(D)**. Coated microtiter plates were incubated with 10 μg/ml hFc or SIGNR3-hFc. Detection was performed using alkaline phosphatase-conjugated goat anti-human-Fc antibody and *p*-nitrophenyl-phosphate. Data are expressed as mean + SEM. The *p*-values were determined using unpaired Student’s *t*-test. Significance is indicated by asterisks (^∗^), ^∗∗∗^*p* ¡ 0.001.

Supplementary Figure S2 **Genotyping of the SIGNR3-gene in wild-type and SIGNR3^−/−^ mice was performed with the following primers (sequences provided from the Consortium for Functional Glycomics):** SD.378 5′-TCCCCCTTCTGCCCTTTTGG-3′, SD.375 5′-CCAATTCCCAGCTTCCACGG-3′, and SD.200 5′-GTTTGGGGGAAATCCAGCTG-3′. Shown is the representative analysis of genomic DNA of wild-type (+/+), heterozygous (+/−), and SIGNR3-deficient (−/−) mice.

## Abbreviations

CD, Crohn’s disease; CLR, C-type lectin receptor; DC, dendritic cell; DCAR, dendritic cell immunoactivating receptor; DC-SIGN, dendritic cell-specific intercellular adhesion molecule-3-grabbing non-integrin; hFc, human Fc; IBD, inflammatory bowel disease; MBL, mannose binding lectin; MGL, macrophage galactose-type lectin; NOD2, nucleotide oligomerization domain 2; PRR, pattern-recognition receptor; SIGNR3, specific intracellular adhesion molecule-3 grabbing non-integrin homolog-related 3; TLR, Toll like receptor; UC, ulcerative colitis.
