# Supplementary material for: The C-Type Lectin Receptor SIGNR3 Binds to Fungi Present in Commensal Microbiota and Influences Immune Regulation in Experimental Colitis
Source: Front Immunol. 2013 Jul 16;4:196. doi: 10.3389/fimmu.2013.00196 (PMC3712271; doi:10.3389/fimmu.2013.00196)
Supplement: Supplementary Figure S1 — Expression and functionality of recombinant SIGNR3-hFc. (A) Expression cassette used for the production of SIGNR3-hFc. The cDNA encoding for the extracellular part of SIGNR3 (SIGNR3 ECD) was amplified using the following primers: SIGNR3 forward 5′-GAATTCCATGCAACTGAAGGCTGAAG-3′ and SIGNR3 reverse 5′ AGATCTTTTGGTGGTGCATGATGAGG-3′. The product was fused in frame to the Fc region of human IgG1. Expression was driven by a hEF1-HTLV promoter and secretion into the culture supernatant was mediated by an external IL2 signal sequence (IL2ss). (B) Western blot analysis of recombinant SIGNR3-hFc. Marker (PageRuler Plus Prestained Protein ladder, Thermo scientific) and 100 ng human Fc (hFc) or SIGNR3-hFc were separated by SDS PAGE, transferred to a nitrocellulose membrane, and detected using an HRP conjugated goat anti-hFc antibody. The calculated molecular weight of the SIGNR3-Fc fusion protein is 44.7 kDa. MGL1-hFc and DCAR-hFc were produced in the same way using target-specific primers. (C,D) Binding of SIGNR3-hFc to immobilized mannan (C) or zymosan (D). Coated microtiter plates were incubated with 10 μg/ml hFc or SIGNR3-hFc. Detection was performed using alkaline phosphatase-conjugated goat anti-human-Fc antibody and p-nitrophenyl-phosphate. Data are expressed as mean + SEM. The p-values were determined using unpaired Student’s t-test. Significance is indicated by asterisks (∗), ∗∗∗p ¡ 0.001. [file 56083_Lepenies_DataSheet1.DOC]

**Supporting information**

**
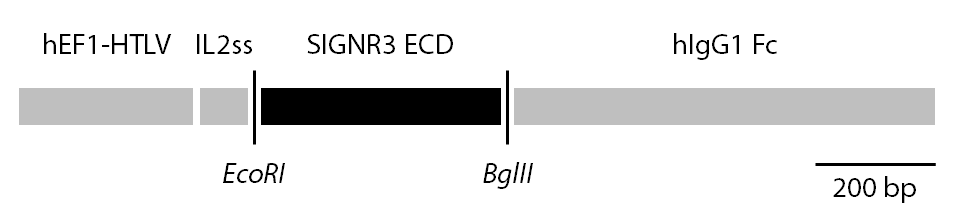
**

**A**

**B**


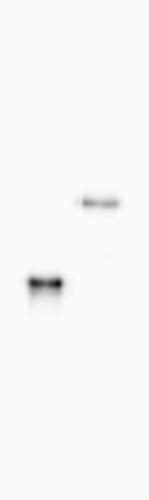


**hFc SR3-hFc**


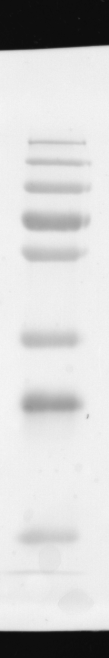


**kDa**

**70**

**55**

**35**

**25**

**15**

**100**

**130**

**250**


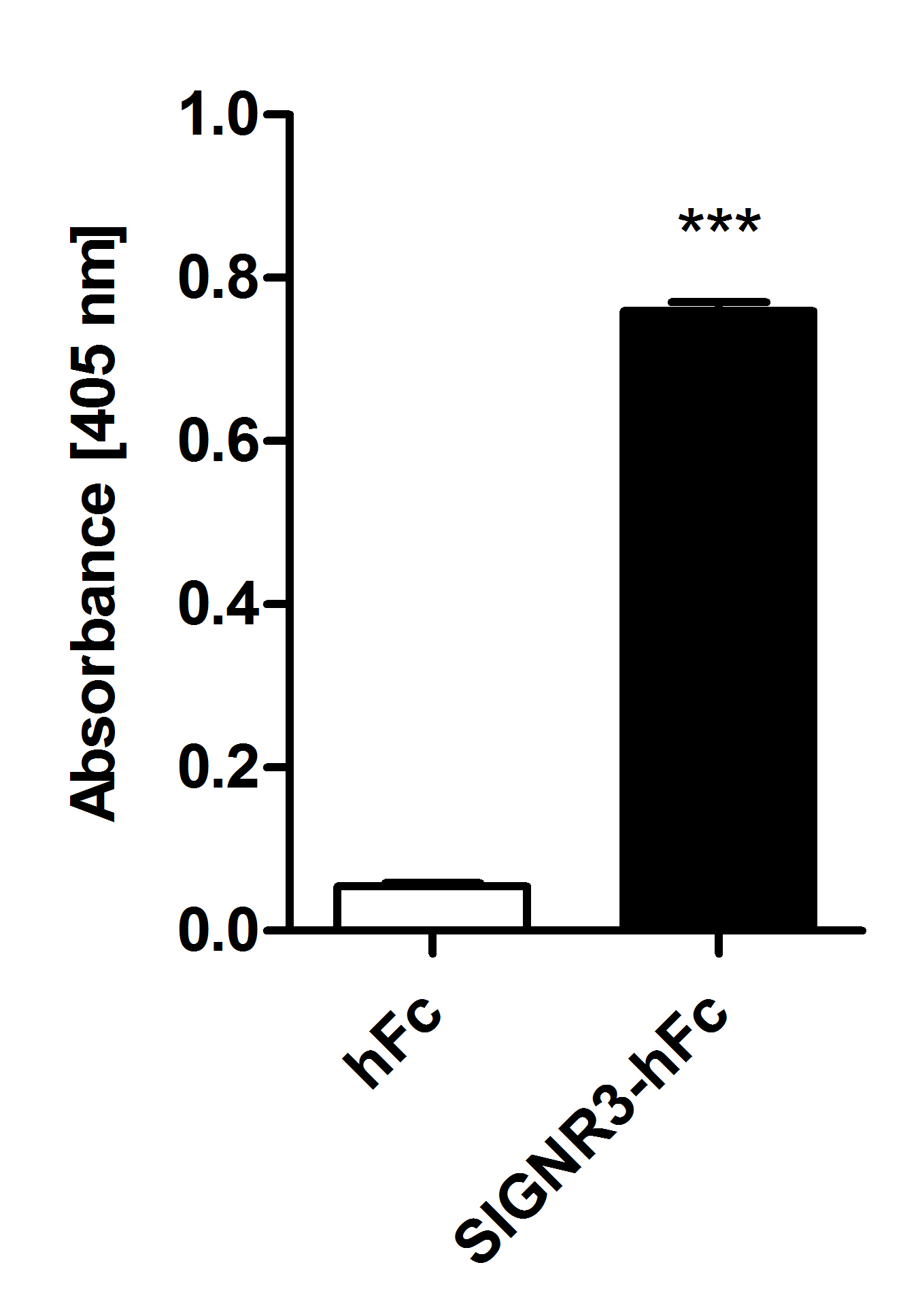


**C**


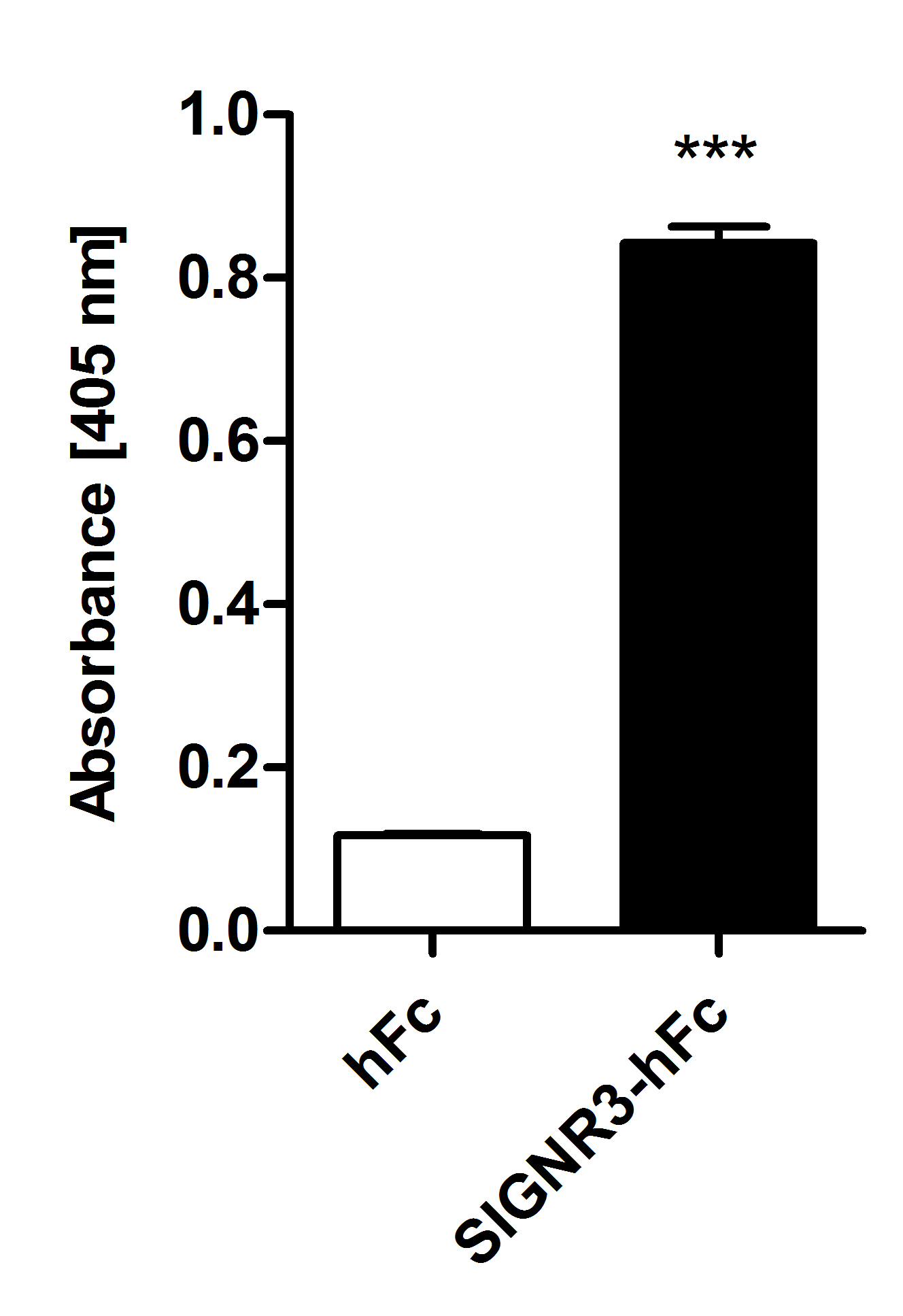


**D**

**Figure S1.** Expression and functionality of recombinant SIGNR3-hFc. **(A)** Expression cassette used for the production of SIGNR3-hFc. The cDNA encoding for the extracellular part of SIGNR3 (SIGNR3 ECD) was amplified using the following primers: SIGNR3 forward 5’-GAATTCCATGCAACTGAAGGCTGAAG-3’ and SIGNR3 reverse 5’‑AGATCTTTTGGTGGTGCATGATGAGG-3’. The product was fused in frame to the Fc region of human IgG1. Expression was driven by a hEF1-HTLV promoter and secretion into the culture supernatant was mediated by an external IL2 signal sequence (IL2ss). **(B)** Western blot analysis of recombinant SIGNR3-hFc. Marker (PageRuler Plus Prestained Protein ladder, Thermo scientific) and 100 ng human Fc (hFc) or SIGNR3-hFc were separated by SDS‑PAGE, transferred to a nitrocellulose membrane and detected using an HRP‑conjugated goat anti-hFc antibody. The calculated molecular weight of the SIGNR3-Fc fusion protein is 44.7 kDa. MGL1‑hFc and DCAR‑hFc were produced in the same way using target-specific primers. **(C, D)** Binding of SIGNR3-hFc to immobilized mannan **(C)** or zymosan **(D)**. Coated microtiter plates were incubated with 10 µg/ml hFc or SIGNR3-hFc. Detection was performed using alkaline phosphatase-conjugated goat anti-human-Fc antibody and p-nitrophenyl-phosphate. Data are expressed as mean + SEM. The p-values were determined using unpaired Student’s t-test. Significance is indicated by asterisks (*), ****p*<0.001.


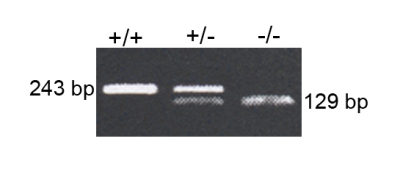


**Figure S2.** Genotyping of the SIGNR3-gene in wild-type and SIGNR3^-/-^ mice was performed with the following primers (sequences provided from the Consortium for Functional Glycomics): SD.378 5’-TCCCCCTTCTGCCCTTTTGG-3’, SD.375 5’-CCAATTCCCAGCTTCCACGG-3’ and SD.200 5’-GTTTGGGGGAAATCCAGCTG-3’. Shown is the representative analysis of genomic DNA of wild-type (+/+), heterozygous (+/-) and SIGNR3 deficient (-/-) mice.
